# Cost-effectiveness analysis of rimegepant for on-demand acute treatment of migraine in China

**DOI:** 10.3389/fneur.2024.1411576

**Published:** 2024-08-23

**Authors:** Shuo Tian, Yuping Yang, Shenglan Tan, Jiani Luo, Chuanyu Yang, Qiao Liu, Yujin Guo

**Affiliations:** ^1^Department of Clinical Pharmacy, Jining First People’s Hospital, Shandong First Medical University, Jining, China; ^2^Department of Pharmacy, The Affiliated Zhuzhou Hospital Xiangya Medical College CSU, Zhuzhou, China; ^3^Department of Pharmacy, The Second Xiangya Hospital of Central South University, Central South University, Changsha, China; ^4^College of Pharmacy, Guilin Medical University, Guilin, China; ^5^Department of Pharmacy, Tongren People’s Hospital, Tongren, China

**Keywords:** cost-effectiveness, rimegepant, migraine, on-demand treatment, China

## Abstract

**Purpose:**

This study assesses the cost-effectiveness of rimegepant for the on-demand acute treatment of migraine in the Chinese population, focusing on headache relief within a 2 h timeframe. Utilizing data from Phase III clinical trials on rimegepant involving Asian populations, this analysis aims to provide essential insights for healthcare decision-making in the context of migraine management in China.

**Patients and methods:**

Employing a decision tree model, this research evaluates the cost-effectiveness of rimegepant over a concise 2 h period, exclusively considering its direct market price of 219.00 CNY per dose for on-demand, single-use treatment upon approval in China. This model is based on pain relief outcomes from a clinical trial, categorizing health outcomes by the achievement of pain freedom and alleviation from the most bothersome symptom within two hours post-administration.

**Results:**

The study unveils that rimegepant adds 0.0018 quality-adjusted life days (QALD) with an incremental cost-effectiveness ratio (ICER) of 122,166.07 CNY/QALD. Against a daily cost-effectiveness threshold derived from the 2023 *per capita* GDP of China (734.45 CNY/day), rimegepant falls short of proving its cost-effectiveness. A significant price reduction to approximately 1.32 CNY per dose is required for rimegepant to be considered cost-effective within this framework. Furthermore, a series of sensitivity analyses were conducted to validate the robustness of these results.

**Conclusion:**

While rimegepant shows clinical efficacy in providing rapid relief from migraine symptoms, its current pricing exceeds the threshold for cost-effectiveness in the Chinese healthcare setting. This study underscores the need for price adjustments to enhance the accessibility and economic viability of new migraine treatments.

## Introduction

Migraine is a prevalent neurological condition characterized by recurrent, severe headaches that significantly impair the quality of life and productivity of those affected (1, 2). In China, the prevalence of migraine is estimated at 9.3%, affecting approximately 130 million people and placing substantial economic and societal burdens on the community (3, 4). The annual direct and indirect costs associated with migraine management exceed 299.4 billion Chinese Yuan (CNY), underscoring the significant economic impact of this condition (5, 6). In China, the current treatment landscape for the acute treatment of migraine primarily involves nonsteroidal anti-inflammatory drugs (NSAIDs, 69%), with ibuprofen being the most commonly used (37%), followed by aspirin (8%), opioids (7%), ergot alkaloids (6%), and triptans (3%) (7). Additionally, many individuals in China opt for herbal medicine to manage migraine symptoms.

Rimegepant, a calcitonin gene-related peptide (CGRP) receptor antagonist, is indicated for the acute treatment of migraine (8). Notably, it also reduces the frequency of migraine recurrences with repeated as-needed use. In the USA, EU, and UK, rimegepant is approved for both the acute treatment of migraine and the preventive treatment of episodic migraine (9, 10). In January 2024, the National Medical Products Administration approved rimegepant in China, but only for the acute treatment of migraine. This approval was based on clinical studies conducted in Asian populations, demonstrating its efficacy in the acute treatment of migraine (11). While studies in USA have shown rimegepant to be effective for the preventive treatment of episodic migraine (12, 13), ongoing research in Chinese populations is still needed to determine its preventive efficacy (11). Mid-term results from the study have been promising, indicating potential benefits in the preventive treatment of migraine for Chinese populations.

Despite its demonstrated efficacy, the accessibility of rimegepant is significantly hindered by its high cost, a critical barrier for the majority of Chinese patients seeking relief from migraine attacks. While Western countries have conducted several cost-effectiveness analyses focusing on the prophylactic use of rimegepant (14), which predominantly yielded negative outcomes due to the price of the drug, there remains a conspicuous void in research pertaining to its cost-effectiveness for the acute treatment of migraine, particularly within the Chinese healthcare landscape.

This study aims to conduct a comprehensive cost-effectiveness analysis of rimegepant for single-use, on-demand acute treatment of migraine among the Chinese demographic (15). By integrating data from clinical trials with the economic realities of the Chinese healthcare environment, this investigation seeks to provide essential insights for healthcare policy-making and decision-making. Beyond offering a novel perspective on patient care, this study aims to influence healthcare policy and the economic evaluation of new treatments, optimizing migraine management strategies in China and improving the lives of those afflicted by this debilitating condition.

## Materials and methods

### Overview

For this economic evaluation, Tree Age Pro software (version 2022, https://www.treeage.com/) was employed to develop the mathematical model that underpins our analysis. The primary aim of the study was to explore the economic and healthcare impacts of introducing rimegepant as an innovative therapeutic option for the on-demand acute treatment of migraine, in comparison to a placebo, specifically within the Chinese healthcare milieu.

Efficacy metrics, including the proportion of individuals achieving headache relief within 2 h, were derived from a targeted Phase III clinical trial within the Asian demographic (11) (ClinicalTrials.gov identifier: NCT04574362, henceforth referred to as the RMG-306 study), alongside a single-dose safety clinical trial (16) (Trial registration: China Center for Drug Evaluation, CTR20210569, henceforth referred to as the RMG-301 study). The reliance on publicly accessible data from these trials meant that our study was exempt from ethical review by the Clinical Ethics Committee of Jining First People’s Hospital, in accordance with the Measures for Ethical Review of Life Science and Medical Research Involving Humans (2023).

This economic analysis was structured to comply with the Chinese guidelines for pharmacoeconomic evaluations (2020) (17), closely adhering to the prescribed methodological framework and analytical standards established for pharmacoeconomic research in China.

### Model construction

In our analysis, a decision tree model was deployed to specifically address the on-demand application of rimegepant for the acute treatment of migraine. This choice of model was predicated on its aptitude for accurately depicting the immediate treatment objectives inherent in migraine management, principally the rapid alleviation of symptoms.

**Figure 1:**
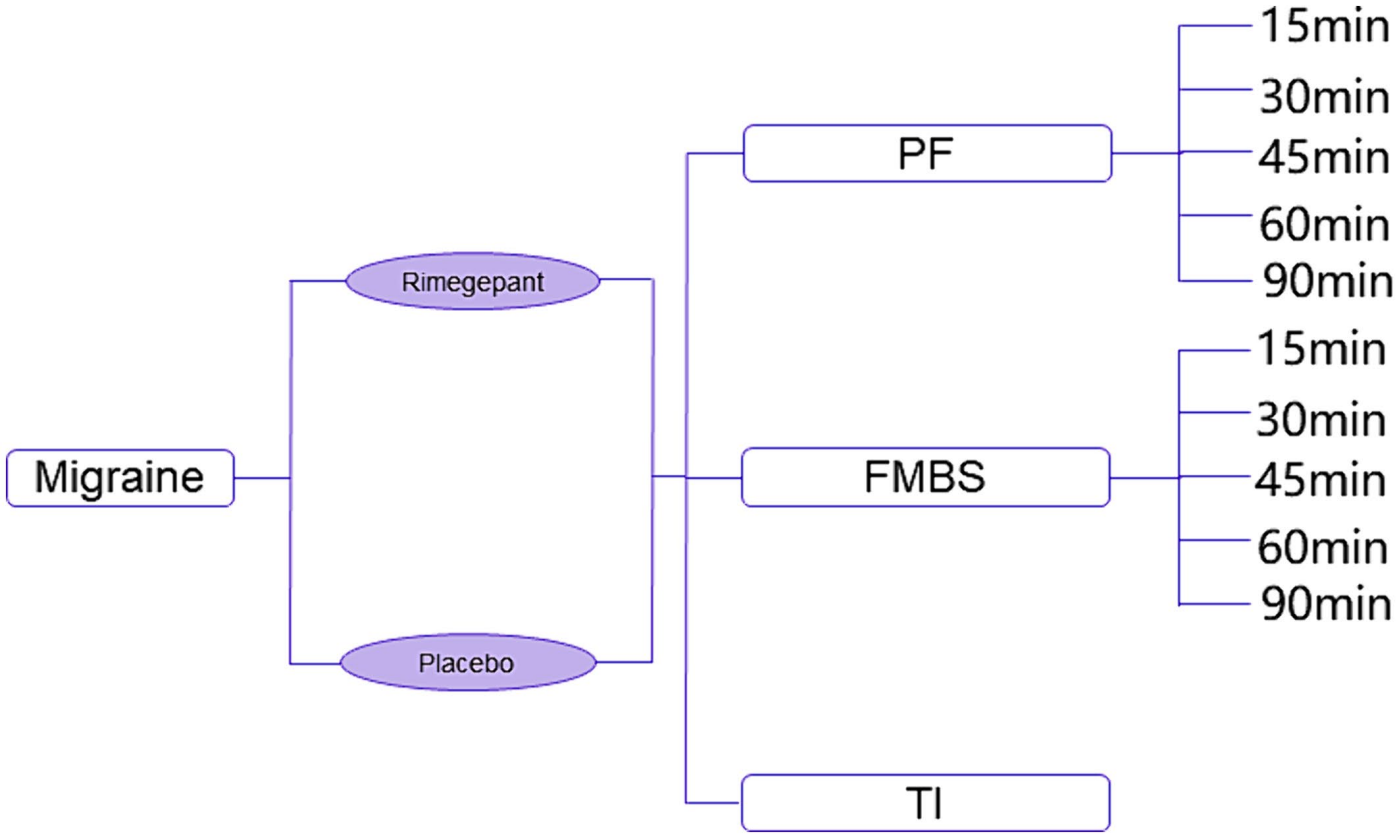
Diagram of decision tree model. PF, pain freedom; FMBS, freedom from most bothersome symptom; TI, treatment ineffectiveness.

In Figure 1, the constructed model identifies three principal health states: pain freedom, freedom from the most bothersome symptoms, and treatment ineffectiveness. For the initial two states, subdivisions were established to mirror the timing of symptom resolution, divided into 15-, 30-, 45-, 60-, 90-, and 120 min intervals post-administration. This segmentation reflects outcomes observed in the RMG-306 study. Based on findings from both the RMG-301 and RMG-306 studies, the incidence of adverse events associated with rimegepant was found to be comparable to that of the placebo group. As a result, our model does not delineate a separate health state for adverse events.

The scenario begins with patients experiencing a migraine, extending over a 2 h period to capture potential developments following the onset of the migraine. This duration reflects the choice between a single dose of rimegepant or a placebo, acknowledging the common tendency among Chinese patients to manage short-lived headache episodes without drug intervention. Owing to the brevity of the analysis period, the study foregoes the application of a discount rate.

Economic evaluation metrics were formulated in accordance with the Chinese pharmacoeconomic evaluation guidelines (2020). Focused on the evaluation of a 2 h acute episode, the willingness-to-pay (WTP) threshold was set at three times the daily-adjusted *per capita* GDP of China for 2023. Treatment efficacy was determined through the computation of cumulative costs, quality-adjusted life days (QALD), and the incremental cost-effectiveness ratio (ICER). A treatment is considered cost-effective if its ICER does not exceed the pre-defined WTP threshold.

### Data and sources

#### Probabilities

The transition probabilities between health states in our model were directly extracted from the RMG-306 study data. The alignment of the health states within the model with the primary and secondary endpoints from the RMG-306 study eliminated the need for data conversion across different follow-up periods.

For the principal health states, occurrence rates were derived from the primary endpoints of the study. Specifically, probabilities for achieving pain freedom or freedom from the most bothersome symptoms 2 h post-administration of rimegepant or placebo were calculated by dividing the number of patients reporting these outcomes by the total number of participants in each respective group. Patients not reaching these states within the defined timeframe were classified under treatment ineffectiveness.

Further subdivisions within these principal states, aimed at capturing more specific timeframes, were based on the secondary outcomes of the study. For example, the probability of achieving pain freedom within 15 min was calculated by the ratio of patients achieving this outcome within 15 min to the total number of patients achieving pain freedom at the 2 hour mark within the same group. Similar calculations were applied for subsequent probability, ensuring the derivation of each minor probability of the state from its proportionate share of patients reaching the primary endpoint at two hours.

Table 1 presents an exhaustive overview of the numerical values of the probabilities and their corresponding mathematical distributions as inputted into our model.

**Table 1:** Probabilities between health states used in the model.

Drug	Rimegepant			Placebo		
Input variable	Base value	Beta distribution		Base value	Beta distribution	
		*α*	*β*		*α*	*β*
Pain freedom 2 h after dosing	0.1982	132	534	0.1068	72	602
Freedom from the MBS 2 h after dosing	0.5045	336	330	0.3576	241	433
Pain freedom 15 min after dosing	0.0379	5	127	0.0972	7	65
Pain freedom 30 min after dosing	0.0152	2	130	0.0417	3	69
Pain freedom 45 min after dosing	0.1136	15	117	0.0694	5	67
Pain freedom 60 min after dosing	0.1742	23	109	0.2083	15	57
Pain freedom 90 min after dosing	0.2803	37	95	0.2500	18	54
Freedom from the MBS 15 min after dosing	0.2024	68	268	0.3112	75	166
Freedom from the MBS 30 min after dosing	0.1310	44	292	0.1037	25	216
Freedom from the MBS 45 min after dosing	0.1458	49	287	0.1411	34	207
Freedom from the MBS 60 min after dosing	0.1369	46	290	0.1369	33	208
Freedom from the MBS 90 min after dosing	0.2054	69	267	0.1950	47	194

MBS, most bothersome symptom; min, minutes; h, hours. (1) Baseline values have been rounded to four decimal places. (2) In the deterministic sensitivity analysis (DSA), the upper and lower limits were set to ±10% of the base value. (3) In the probabilistic sensitivity analysis (PSA), the probabilities were determined using the beta distribution values obtained from the specified *α* and *β* parameters.

#### Utilities

Due to the lack of direct quality of life research associated with the RMG-306 study for the acute treatment of migraine, the utility values for our study were sourced from existing cost-effectiveness analyses and the reports by the Institute for Clinical and Economic Review on migraine^1^ (18).

Originally, these utility values (19) were calculated based on the number of monthly headache days (MHD) to estimate quality-adjusted life years (QALYs). For the purpose of our study, which assesses the impact of a 2 h migraine episode, these values were proportionally adjusted to quality-adjusted life days (QALD). This adjustment involves translating the data from a 30 day basis to a 2 h period to reflect the impact on quality of life during migraine episodes. In addition, for the health state associated with achieving freedom from the most bothersome symptoms, the utility estimation was specifically adjusted by applying a factor of 0.87 to the utility value for the pain freedom state (18). Table 2 provides a detailed presentation of the utility values employed in our model, including their mathematical distributions.

**Table 2:** Utility values for health states.

Health state	Base value	Low value	High value	SD
Pain freedom 15 min after dosing	0.7573	0.7194	0.7952	0.1662
Pain freedom 30 min after dosing*	0.6449	0.6127	0.6771	0.2817
Pain freedom 45 min after dosing*	0.6764	0.6426	0.7102	0.2458
Pain freedom 60 min after dosing	0.6420	0.6010	0.6741	0.2543
Pain freedom 90 min after dosing	0.5916	0.5620	0.6212	0.2549
Pain freedom 120 min after dosing	0.5040	0.4789	0.5292	0.2835
Sustained pain in 2 h	0.4400	0.3740	0.5020	0.2477

SD, standard deviation; min, minutes; h, hours. (1) In the PSA, the probabilities were determined using the beta distribution values obtained from the specified base value and SD parameters. (2) *The higher health utility value associated with pain freedom 45 min after dosing compared to 30 min is based on data from a referenced study. Despite its deviation from common expectations, these values are reflective of the original dataset provided in the cited literature. (3) The data presented in this table represent unprocessed raw health utility values (in QALY) for headache occurrences. These values undergo transformation to QALD before being inputted into the research model.

#### Cost

In our analysis, the drug costs are exclusively associated with the price of a single, on-demand dose of rimegepant, as the study does not consider the financial implications of adverse events. The standard dosage for rimegepant is set at 75 mg per administration, with a recommended limit of no more than one dose per day. Accordingly, our model includes the cost for one dose of rimegepant, reflecting its pricing strategy following its recent introduction to the Chinese market. Table 3 presents a detailed presentation of the cost data, including the average price, minimum price, maximum price, and standard deviation, along with the mathematical distribution for the price of rimegepant.

**Table 3:** Drug dose and costs.

Drug (CNY)	Dose	Base value price	Low value price	High value price	SD*
Rimegepant	75 mg/once	219.00	175.20	262.80	22.24
Placebo	–	0	0	0	0

CNY, Chinese Yuan; SD, standard deviation. (1) In the PSA, the probabilities were determined using the Gamma distribution values obtained from the specified base value and SD parameters. (2)* The SD of drug prices is derived from a comprehensive market survey conducted within the local price market.

### Sensitivity analysis

To assess the stability and reliability of our model in the face of parameter uncertainties, we conducted both one-way deterministic sensitivity analysis (DSA) and probabilistic sensitivity analysis (PSA). The DSA explored how variations in each individual parameter influenced the overall cost-effectiveness outcomes of the model. Parameter ranges for the DSA were derived from existing literature where possible, or otherwise set to ±10% of the base-case values to account for potential fluctuations. The PSA, on the other hand, was implemented to examine the collective impact of multiple parameter changes on the cost-effectiveness findings. This comprehensive analysis was facilitated by conducting a Monte Carlo simulation with 100 iterations, producing a range of 100 ICER estimates for rimegepant compared to the control group. Detailed descriptions of the ranges and mathematical distributions applied to each parameter in both the DSA and PSA can be found in Tables 1–3.

## Results

### Base-case analysis

In our study, a 2 h assessment was carried out to compare the on-demand acute treatment efficacy of rimegepant for migraine against a placebo. The incremental effectiveness attributed to rimegepant was determined to be 0.0018 QALD, with rimegepant demonstrating an effectiveness of 0.0438 compared to 0.0420 for the placebo. The administration of rimegepant was associated with an incremental cost of 219.00 CNY, resulting in an ICER of 122,166.07 CNY/QALD. Compared to the WTP threshold, established at three times the daily-adjusted 2023 *per capita* GDP of China (734.45 CNY), the ICER for rimegepant substantially surpasses this threshold. Our analysis further indicated that for rimegepant to align with the defined WTP threshold, a price reduction to 1.32 CNY or less would be necessary. The detailed outcomes of our base-case analysis are meticulously cataloged in Table 4.

**Table 4:** Base case results.

Drug	Cost	Effectiveness	ICER (CNY/QALD)
Placebo	0	0.0420	
Rimegepant	219.00	0.0438	122166.07
Rimegepant (at a reduced price)	1.32	0.0438	734.45

ICER, incremental cost-effectiveness ratio; CNY, Chinese Yuan; QALD, quality-adjusted life days.

### Sensitivity analyses

**Figure 2:**
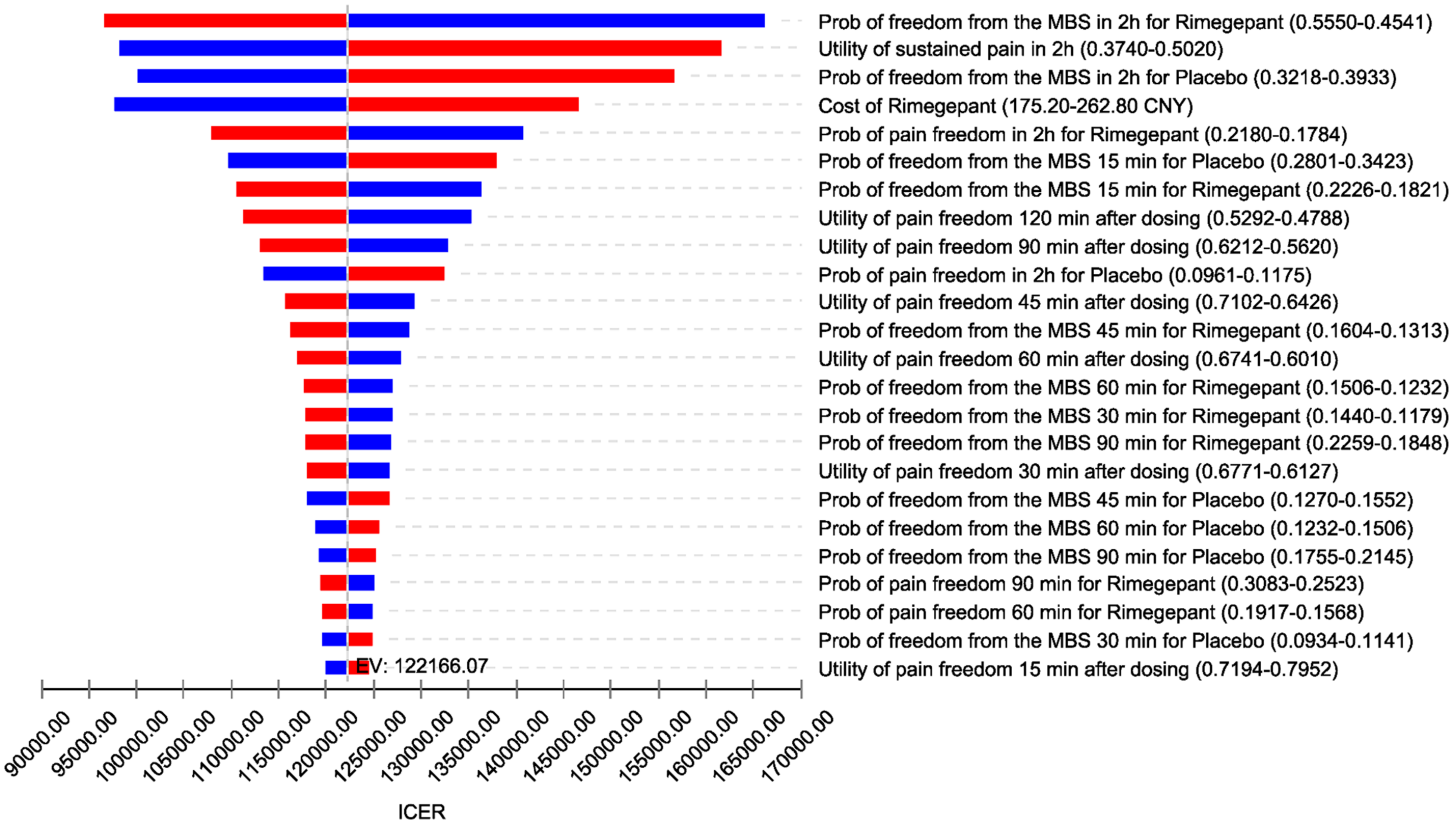
Tornado diagram (deterministic sensitivity analysis results). ICER, incremental cost-effectiveness ratio; EV, expected value; CNY, Chinese Yuan; Prob, probabilities; MBS, most bothersome symptom; min, minutes; h, hours.

The results of the DSA are depicted in Figure 2, utilizing a tornado diagram. This diagram elucidates the impact of individual model parameter variations on the ICER. Notably, the transition probabilities to achieving freedom from the most bothersome symptoms within the rimegepant exerted the greatest influence on the ICER, followed by the utility value representing no improvement in migraine symptoms after a 2 h period. Additionally, the transition probabilities to achieving freedom from the most bothersome symptoms within the placebo emerged as the third most influential factor. Subsequently, the cost of rimegepant also significantly affected the ICER. It is worth noting that the stability of DSA results in our study may be attributed to the decrease in numerical values after utility value transformation to QALD in our research methodology. The expected value (EV) line, representing the base-case analysis outcome of 122,166.07 CNY/QALD, serves as a reference for the baseline result. Increases in model inputs are depicted by red bars, while decreases are indicated by blue bars, with each bar reflecting the ICER range derived from varying each parameter within set boundaries.

**Figure 3:**
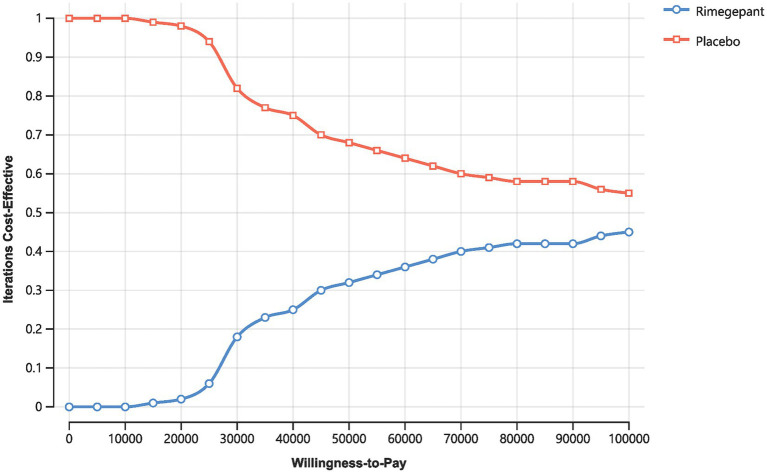
Acceptability curve.

Figure 3 displays the cost-effectiveness acceptability curve, indicating the probability of rimegepant being cost-effective across various WTP thresholds. Notably, at a WTP threshold below 30,000 CNY, the likelihood of rimegepant being cost-effective is substantially low, with only a 18% probability at a WTP of 30,000 CNY, as opposed to 82% for the placebo. However, even as the WTP threshold rises, the probability of rimegepant being cost-effective witnesses only slight increases, reaching a mere 45% at a WTP of 100,000 CNY, while the probability for the placebo diminishes to 55%.

**Figure 4:**
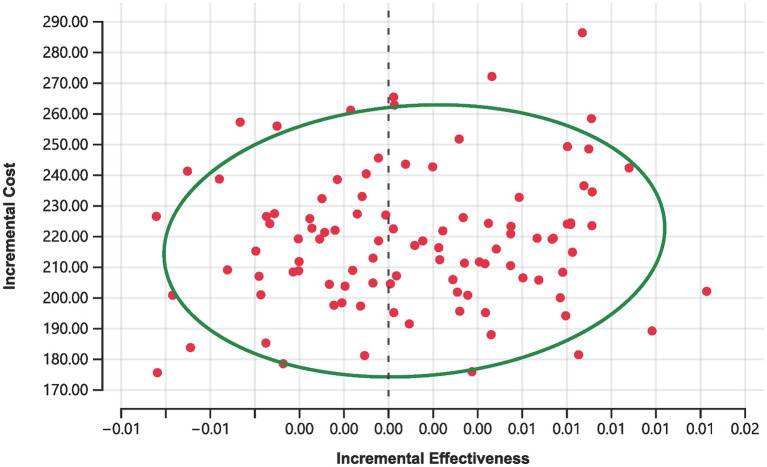
Scatter plot for rimegepant.

Moreover, Monte Carlo simulations depicted in Figure 4 offer further insights. Out of 100 simulations conducted at the current price of rimegepant, none achieved cost-effectiveness, with 44% indicating a complete absence of cost-effectiveness (i.e., increased costs with diminished effectiveness relative to the placebo). The remaining 56% of simulations resulted in ICER surpassing the established WTP threshold of 734.45 CNY.

## Discussion

Rimegepant has secured approval for migraine treatment and is recommended in migraine management guidelines in the United States (9), Europe (20), and China (15) albeit with its application in Chinese guidelines specifically confined to migraine management. It has been evaluated in several Phase III clinical trials internationally, addressing both treatment and prevention, and showing positive efficacy in populations across the United States (12, 13) and Asia (11), including China and Korea. Nonetheless, cost-effectiveness analyses from the U.S. perspective suggest the potential limitations of rimegepant in being cost-effective for migraine prevention, with its value for acute treatment also appearing comparatively lower (14).

Conducting a cost-effectiveness analysis of rimegepant for the treatment of migraine in China marks a novel and critical exploration, essential for appraising its financial feasibility within the Chinese healthcare landscape. Leveraging existing cost-effectiveness studies (14, 18) and quality of life research (21–23) on rimegepant, our study endeavors to bridge this gap, aiming to provide evidence-based treatment insights for migraine patients in China. Additionally, our investigation accounts for the correlation between Chinese *per capita* GDP and the costs associated with treatment, aiming to ensure that our findings are pragmatically relevant and regionally tailored.

Our study presents several key strengths. Firstly, the data on headache resolution rates within 2 h post-rimegepant administration, derived directly from the RMG-306 study—which specifically investigates migraine episodes among the Asian population—ensures an accurate depiction of real-world outcomes. Secondly, we employ an innovative approach to utility value estimation in the face of limited data availability. By adapting utility values, originally based on the number of MHD, to assess acute episodes within a concise 2 h timeframe, our study adeptly addresses the complexities involved in translating chronic condition utilities to the acute setting. Thirdly, although rimegepant has only recently been introduced to the Chinese market, leading to relatively stable pricing, our model anticipates potential future pricing variations by incorporating a 20% margin of variability in the cost rimegepant. Lastly, while our results concur with studies from the United States indicating that rimegepant lacks cost-effectiveness, our analysis goes further by determining a reference price point at which rimegepant could achieve cost-effectiveness within the Chinese context. Despite the stark contrast between our proposed price reference and the current pricing of rimegepant, it is important to note that in real-world scenarios, the majority of migraine patients often opt for transient endurance or choose to take a single dose of nonsteroidal anti-inflammatory drugs such as ibuprofen. Moreover, within the context of Chinese government-led centralized drug procurement policies, our suggested price reference remains practical and relevant.

Our study encounters several limitations that merit consideration. Firstly, our analysis is confined to the single, on-demand use scenario of rimegepant, not accounting for the recurrent nature of migraine attacks that many patients experience. Unlike its approval in Western countries for both acute and preventive treatment, rimegepant in China is only approved for the acute treatment of migraine. This limitation overlooks the long-term economic and clinical implications of repeated migraine episodes and the potential cumulative benefit of treatment. Given the high cost of rimegepant and its impact on patient compliance, our analysis, although based on a single intake, still holds significant reference value. Secondly, the innovative methodology of deriving utility values from the number of MHD to assess a 2 h episode migraine might not fully capture the nonlinear impact short-term events could have on quality of life. This adaptation, while practical in the absence of specific utility data, introduces a degree of speculation regarding the true quality of life impact during such episodes. Sensitivity analyses indicate that our method of handling utility data may contribute to model instability. However, this instability does not affect the ultimate negative findings. In both DSA and PSA, within the predefined parameter ranges and distributions, the current pricing of rimegepant does not demonstrate cost-effectiveness. Thirdly, the cost considerations in our model are narrowly focused on the price of rimegepant alone. Given the 2 h time frame of our study, we did not incorporate the broader economic losses attributed to ongoing migraine episodes, such as reduced productivity or additional healthcare utilization. Including these broader economic impacts could potentially shift the cost-effectiveness balance more favorably. Lastly, while rimegepant has been demonstrated to be safe with a low incidence of adverse events across multiple clinical trials (11–13), our model does not account for the occurrence of such events. The exclusion of adverse event considerations may skew our cost-effectiveness analysis, underestimating the true cost and overestimating the value associated with using rimegepant for the acute treatment of migraine.

## Conclusion

Within the context of Chinese economic landscape and the current market pricing of rimegepant, our study conclusively finds that the on-demand, single-use of rimegepant for the acute treatment of migraine does not demonstrate cost-effectiveness. By highlighting the economic limitations of rimegepant application under its current pricing, our analysis underscores the need for price adjustments or alternative strategies to enhance its cost-effectiveness. Our findings could inform future revisions of treatment guidelines and healthcare policies, providing critical insights for healthcare decision-making concerning the acute treatment of migraine management in China.

## Data Availability

The original contributions presented in the study are included in the article/supplementary material, further inquiries can be directed to the corresponding authors.

## Footnotes

^1^ https://icer.org/assessment/acute-migraine-2020/

## References

[1] Lipton RB Croop R Stock EG Stock DA Morris BA Frost M. Rimegepant, an Oral calcitonin gene-related peptide receptor antagonist, for migraine. N Engl J Med. (2019) 381:142–9. doi: 10.1056/NEJMoa1811090 31291516

[2] Slomski A. Oral Rimegepant safe, effective for migraine prevention. JAMA. (2021) 325:713. doi: 10.1001/jama.2021.0651, PMID: 33620400

[3] Takeshima T Wan Q Zhang Y Komori M Stretton S Rajan N. Prevalence, burden, and clinical management of migraine in China, Japan, and South Korea: a comprehensive review of the literature. J Headache Pain. (2019) 20:111. doi: 10.1186/s10194-019-1062-4, PMID: 31805851 PMC6896325

[4] Stovner LJ Hagen K Linde M Steiner TJ. The global prevalence of headache: an update, with analysis of the influences of methodological factors on prevalence estimates. J Headache Pain. (2022) 23:34. doi: 10.1186/s10194-022-01402-2, PMID: 35410119 PMC9004186

[5] Ashina M Katsarava Z Do TP Buse DC Pozo-Rosich P Özge A. Migraine: epidemiology and systems of care. Lancet. (2021) 397:1485–95. doi: 10.1016/s0140-6736(20)32160-7 33773613

[6] Yao C Wang Y Wang L Liu Y Liu J Qi J. Burden of headache disorders in China, 1990-2017: findings from the global burden of disease study 2017. J Headache Pain. (2019) 20:102. doi: 10.1186/s10194-019-1048-2, PMID: 31699022 PMC6836347

[7] Yu S Zhang Y Yao Y Cao H. Migraine treatment and healthcare costs: retrospective analysis of the China health insurance research association (CHIRA) database. J Headache Pain. (2020) 21:53. doi: 10.1186/s10194-020-01117-2, PMID: 32404048 PMC7222520

[8] Dos Santos JBR da Silva MRR. Small molecule CGRP receptor antagonists for the preventive treatment of migraine: a review. Eur J Pharmacol. (2022) 922:174902. doi: 10.1016/j.ejphar.2022.174902 35358493

[9] Ailani J Burch RC Robbins MS. The American headache society consensus statement: update on integrating new migraine treatments into clinical practice. Headache. (2021) 61:1021–39. doi: 10.1111/head.14153, PMID: 34160823

[10] Scott L. J. Rimegepant: first approval. Drugs. (2020) 80:741–6. doi: 10.1007/s40265-020-01301-3 32270407

[11] Yu S Kim BK Guo A Kim MH Zhang M Wang Z. Safety and efficacy of rimegepant orally disintegrating tablet for the acute treatment of migraine in China and South Korea: a phase 3, double-blind, randomised, placebo-controlled trial. Lancet Neurol. (2023) 22:476–84. doi: 10.1016/s1474-4422(23)00126-6, PMID: 37210098

[12] Croop R Goadsby PJ Stock DA Conway CM Forshaw M Stock EG. Efficacy, safety, and tolerability of rimegepant orally disintegrating tablet for the acute treatment of migraine: a randomised, phase 3, double-blind, placebo-controlled trial. Lancet. (2019) 394:737–45. doi: 10.1016/s0140-6736(19)31606-x 31311674

[13] Croop R Lipton RB Kudrow D Stock DA Kamen L Conway CM. Oral rimegepant for preventive treatment of migraine: a phase 2/3, randomised, double-blind, placebo-controlled trial. Lancet. (2021) 397:51–60. doi: 10.1016/s0140-6736(20)32544-7 33338437

[14] Thaliffdeen R Yu A Rascati K. Cost-effectiveness evaluation of Oral CGRP antagonists, Atogepant and Rimegepant, for the preventative treatment of episodic migraine: results from a US societal perspective model. Clin Drug Investig. (2024) 44:209–17. doi: 10.1007/s40261-024-01345-3, PMID: 38381352

[15] Chinese Medical Association Group. Guide to the prevention and treatment of migraine in China [zhong guo pian tou tong fang zhi zhi nan]. Chin Med News. (2023) 38:8. doi: 10.3760/cma.j.issn.1000-8039.2023.12.113

[16] Li Y Wang X Ballesteros-Perez A Bertz R Lu Z. Pharmacokinetics and safety of single and multiple daily dosing of 75-mg Rimegepant orally disintegrating tablets in healthy Chinese adults: a randomized placebo-controlled trial. Clin Pharmacol Drug Dev. (2023) 12:594–601. doi: 10.1002/cpdd.1230 36808268

[17] Yue X Li Y Wu J Guo JJ. Current development and practice of Pharmacoeconomic evaluation guidelines for universal health coverage in China. Value Health Regional Issues. (2021) 24:1–5. doi: 10.1016/j.vhri.2020.07.580, PMID: 33349598

[18] Agboola F Atlas SJ Touchette DR Borrelli EP Rind DM Pearson SD. The effectiveness and value of novel acute treatments for migraine. J Manag Care Spec Pharm. (2020) 26:1456–62. doi: 10.18553/jmcp.2020.26.11.1456, PMID: 33119447 PMC10391055

[19] Mistry H Naghdi S Underwood M Duncan C Madan J Matharu M. Competing treatments for migraine: a headache for decision-makers. J Headache Pain. (2023) 24:162. doi: 10.1186/s10194-023-01686-y, PMID: 38053051 PMC10696771

[20] Eigenbrodt AK Ashina H Khan S Diener HC Mitsikostas DD Sinclair AJ. Diagnosis and management of migraine in ten steps. Nat Rev Neurol. (2021) 17:501–14. doi: 10.1038/s41582-021-00509-5, PMID: 34145431 PMC8321897

[21] Tassorelli C Onishchenko K Halker Singh RB Duan M Dupont-Benjamin L Hemstock M. Comparative efficacy, quality of life, safety, and tolerability of atogepant and rimegepant in migraine prevention: a matching-adjusted indirect comparison analysis. Cephalalgia Int J Headache. (2024) 44:3331024241235156. doi: 10.1177/03331024241235156, PMID: 38410850

[22] Popoff E Johnston K Croop R Thiry A Harris L Powell L. Matching-adjusted indirect comparisons of oral rimegepant versus placebo, erenumab, and galcanezumab examining monthly migraine days and health-related quality of life in the treatment of migraine. Headache. (2021) 61:906–15. doi: 10.1111/head.14128, PMID: 34021585 PMC8361942

[23] Johnston K Harris L Powell L Popoff E Coric V L’Italien G. Monthly migraine days, tablet utilization, and quality of life associated with Rimegepant—post hoc results from an open label safety study (BHV3000-201). J Headache Pain. (2022) 23:10. doi: 10.1186/s10194-021-01378-5, PMID: 35038983 PMC8903552

